# The use of a portable metabolic monitoring device for measuring RMR in healthy adults

**DOI:** 10.1017/S0007114520001014

**Published:** 2020-12-14

**Authors:** Suey S. Y. Yeung, Marijke C. Trappenburg, Carel G. M. Meskers, Andrea B. Maier, Esmee M. Reijnierse

**Affiliations:** 1 Department of Human Movement Sciences, @AgeAmsterdam, Vrije Universiteit Amsterdam, Amsterdam Movement Sciences, Amsterdam, The Netherlands; 2 Department of Medicine and Aged Care, @AgeMelbourne, The Royal Melbourne Hospital, The University of Melbourne, Melbourne, VIC, Australia; 3 Department of Internal Medicine, Section of Gerontology and Geriatrics, Amsterdam UMC, Vrije Universiteit Amsterdam, Amsterdam, The Netherlands; 4 Department of Internal Medicine, Amstelland Hospital, Amstelveen, The Netherlands; 5 Department of Rehabilitation Medicine, Amsterdam UMC, Vrije Universiteit Amsterdam, Amsterdam, The Netherlands

**Keywords:** RMR, Basal metabolism, Indirect calorimetry, Nutrition assessment

## Abstract

Objective measurement of RMR may be important for optimal nutritional care but is hindered by the price and practicality of the metabolic monitoring device. This study compared two metabolic monitoring devices for measuring RMR and VO_2_ and compared the measured RMR with the predicted RMR calculated from equations. RMR was measured using QUARK RMR (reference device) and Fitmate GS (COSMED) in a random order for 30 min, each on fasted participants. In total, sixty-eight adults participated (median age 22 years, interquartile range 21–32). Pearson correlation showed that RMR (*r* 0·86) and VO_2_ (*r* 0·86) were highly correlated between the two devices (*P* < 0·05). Intraclass correlation coefficients (ICC) showed good relative agreements regarding RMR (ICC = 0·84) and VO_2_ (ICC = 0·84) (*P* < 0·05). RMR measured by QUARK RMR was significantly higher (649 (sd 753) kJ/d) than Fitmate GS. Equations significantly overpredicted RMR. Accurate RMR (i.e. within ±10 % of the RMR measured by QUARK RMR) was found among 38 % of the participants for Fitmate GS and among 46–68 % depending on the equations. Bland–Altman analysis showed a low absolute agreement with QUARK RMR at an individual level for both Fitmate GS (limits of agreement (LOA): −828 to +2125 kJ/d) and equations (LOA ranged from −1979 to +1879 kJ/d). In conclusion, both Fitmate GS and predictive equations had low absolute agreements with QUARK RMR at an individual level. Therefore, these limitations should be considered when determining RMR using Fitmate GS or equations.

RMR represents the amount of energy needed to sustain physiological function at rest^(1)^. Predictive equations are widely used in clinical practice to estimate the RMR of individuals^(2)^. These equations account for factors such as age, sex, height, weight and body composition; however, they do not account for other factors such as physical activity and health status that are known to affect the RMR^(3,4)^. Therefore, these equations can significantly overestimate or underestimate the RMR^(4,5)^. Accurate and objective measurement of the RMR is important for optimal nutritional care and improvement of health outcomes such as preventing unintentional weight loss or gaining weight, fewer complications and shorter length of hospital stay^(6–8)^. Therefore, RMR should likely be measured objectively rather than predicted to determine the energy needs of individuals^(2)^.

Metabolic monitoring devices such as QUARK RMR (COSMED) are however relatively expensive, less portable and complicated to calibrate and use, making routine RMR measurement difficult and impractical. A new portable device, Fitmate GS (COSMED), is easier to calibrate, is portable, requires less space and less intensive training for operators and has lower operational costs. QUARK RMR has been validated against the ‘gold standard’ DELTATRAC (no longer in production) in healthy adults^(9)^. Fitmate GS has also been validated against the ‘gold standard’ in hospitalised patients^(10)^, but not in healthy adults. Although Fitmate GS has been validated in healthy adults against Douglas bag^(11)^, which has been considered by some researchers as the ‘gold standard’ against which a new device is evaluated, yet formal validation of the Douglas bag has not been carried out^(12)^. Therefore, it is of interest to compare the RMR measurement among healthy adults between Fitmate GS with the previously validated QUARK RMR. In addition, both metabolic monitoring devices have not been investigated yet in the same individuals.

The aims of this study were (1) to compare QUARK RMR and Fitmate GS with canopy hood for measuring RMR and VO_2_ in healthy adults and (2) to compare the measured RMR with the RMR calculated from predictive equations.

## Methods

### Study design

This cross-sectional study included sixty-eight healthy adults recruited based on email invitations sent to the staff and students and by the word-of-mouth method. The inclusion criterion was an age of 18 years and over. Exclusion criteria encompassed pregnancy, lactation or suffering any of the following diseases: metabolic diseases (insulin-dependent), cancer, chronic obstructive pulmonary disease, rheumatoid arthritis, liver disease and cardiac failure with a pacemaker. For interested participants, the researchers provided a detailed information letter and consent form. Following this, the researchers checked the inclusion and exclusion criteria by telephone or in-person. The protocol was explained prior to the experiment.

This study was conducted according to the guidelines laid down in the Declaration of Helsinki and was approved by the Melbourne Health Human Research Ethics Committee, Melbourne, Victoria, Australia (HREC/16/MH/346). All participants gave written informed consent.

### Sample size calculation

The mean RMR in healthy adults was previously reported to be 6987 (sd 1410) kJ/d using the QUARK RMR and 6866 (sd 1251) kJ/d using the DELTATRAC II system (Sensormedics)^(9)^. Based on the literature and our hypothesised mean difference, a sample size of thirty-one participants would be required to detect a mean difference of 628 kJ/d of RMR with an sd of 1255 kJ/d, a statistical power of 0·80 and an α level of 0·05.

### Study procedure

Before the measurement, participants were instructed to refrain from moderate to vigorous physical activity for 24 h, to be fasted overnight for at least 7 h and abstain from nicotine products for at least 2·5 h^(13)^. Compliance to these instructions was verified on the day of the measurement by asking the participants if they followed the instructions and tests were rescheduled if non-compliant (*n* 2). Participants completed a short questionnaire assessing sex, age, medical history, number of medications used and physical activity. The International Physical Activity Questionnaire was used to assess the participants’ physical activity status over the past 7 d before the test^(14)^. Physical activity level was categorised into high, moderate and low according to the scoring protocol which takes intensity, frequency and duration into account^(15)^. Body weight was measured to the nearest 0·1 kg, and height was measured to the nearest 0·1 cm using a weighing scale and stadiometer, respectively. BMI was calculated by weight in kg divided by height in square meters. Direct-segmental multi-frequency bioelectrical impedance analysis (DSM-BIA; InBody S10; Biospace Co. Ltd) was used to measure body composition including skeletal muscle mass (expressed in kg), fat-free mass (FFM, expressed in kg) and fat mass (expressed in %). RMR (in kJ/d) was measured using two metabolic monitoring devices: the QUARK RMR and Fitmate GS systems in a pre-set random order to avoid confounding due to order effects. Randomisation was performed using the allocation ratio of 1:1. The research team prepared an envelope which included sixty-eight cards (thirty-four printed ‘QUARK RMR’ and thirty-four printed ‘Fitmate GS’), to randomise the order of testing the devices^(16)^. Participants were asked to pick a card from the envelope randomly.

The metabolic monitoring devices were calibrated according to the manufacturers’ guidelines before each measurement. After completing the calibration procedure, the canopy hood was placed over the participants’ head. Each RMR measurement was conducted for 30 min^(4,17)^, while participants were in a supine position and instructed to limit movement, talking and to avoid sleeping. Participants were urged to indicate if any discomfort was experienced during the RMR measurement by raising their hand. After the first RMR measurement, participants were given a standardised break in which they were instructed to take a walk over a standardised trajectory at their usual pace for 10 min to interrupt the steady state achieved in the first RMR measurement and to align pre-test conditions (i.e. no prior resting period)^(18,19)^ for the measurements with the second device. The first 5 min of the data from both the measurements was discarded because the participants had to adapt their breathing while wearing the canopy hood^(13,20)^. Data of RMR and VO_2_ between 5 and 30 min were averaged and used in the analysis. All participants were measured by the same researcher (S. S. Y. Y.).

### QUARK RMR

The QUARK RMR (COSMED) is a metabolic monitoring device operated with an external power supply only and should be connected to a computer, monitor and printer for displaying the RMR and VO_2_ readings during the measurement and printing the report. A protocolled methanol burning test^(21,22)^ performed prior to this study by the manufacturer revealed a RQ (the ratio between the produced carbon dioxide and the oxygen consumed) of 0·68 which was within the acceptable range of 0·64–0·69^(21,23)^. The QUARK RMR has been validated against the ‘gold standard’ DELTATRAC in healthy adults^(9)^ and was considered as the reference device in this study.

On each measurement day, 5 min warm-up time of the system was required according to the manufacturer’s instructions before the calibration. Before the measurement, the researcher performed a flowmeter and gas calibration. For the flowmeter calibration, a calibration syringe was connected to a flowmeter and the syringe pistol was moved slowly in and out until the measured inspiratory and expiratory volumes were displayed on the screen indicating the end of the calibration. For gas calibration, the sampling line was disconnected from the flowmeter and connected to the front panel of the QUARK RMR. The output pressure of the oxygen cylinder was adjusted according to the ranges of output pressure, as shown on the screen. The QUARK RMR contains both oxygen (VO_2_) and carbon dioxide (VCO_2_) sensors, and therefore it directly measures the actual RQ. Steady state was achieved for QUARK RMR when the CV in VO_2_ and VCO_2_ were both <10 % during the 30-min measurement (discarding the first 5 min).

### Fitmate GS

The Fitmate GS (COSMED) is a portable desktop metabolic monitoring device. It is battery-operated, weighs 1·5 kg and has a built-in colour display and graphics printer. No warm-up time is required. Before each measurement, the researcher performed a flowmeter calibration. A calibration syringe was connected to the flowmeter, and the syringe pistol was moved slowly in and out until the results of the calibration were displayed on the screen. After that, an automatic O_2_ analyser calibration preceded the beginning of the measurement. The Fitmate GS system does not contain a carbon dioxide sensor, so it calculates the RMR by estimating carbon dioxide production from a fixed RQ of 0·85 based on the abbreviated Weir equation^(24)^: (3·9 × (VO_2_) + 1·1 × (RQ × VO_2_)) × 1·44. The steady state was achieved for Fitmate GS when the CV in VO_2_ was <10 % during the 30-min measurement (discarding the first 5 min).

### Predictive equations

The RMR measured by the two metabolic monitoring devices were compared with predicted RMR calculated by commonly used predictive equations^(2,25)^. Weight-based equations encompassed Harris & Benedict^(26)^ (age, sex, weight and height), Mifflin *et al.*^(27)^ (age, sex, weight and height), Muller *et al.*^(28)^ (age, sex, weight), Owen *et al.*^(29,30)^ (sex and weight), Henry^(31)^ (age, sex and weight), Henry^(31)^ (age, sex, weight and height), Schofield^(32)^ (age, sex and weight), Schofield^(32)^ (age, sex, weight and height), WHO^(33)^ (age, sex and weight) and WHO^(33)^ (age, sex, weight and height). FFM-based equations encompassed Mifflin *et al.*^(27)^ (FFM), Muller *et al.*^(28)^ (age, sex, FFM, FM) and Owen *et al.*^(29,30)^ (sex, FFM).

### Statistical analysis

Continuous variables with a normal distribution were presented as mean values and standard deviations, while those with a non-normal distribution were presented as medians and interquartile ranges (IQR). Categorical variables were presented as numbers and percentages.

The number (%) of participants with and without steady state was calculated. Participants with steady state were defined as achieving a steady state for both QUARK RMR and Fitmate GS, while participants without steady state were defined as not achieving a steady state for QUARK RMR and/or Fitmate GS. Analysis stratified by participants with and without steady state, next to a pooled analysis, was conducted. Paired sample *t* tests were used to compare mean differences in the RMR and VO_2_ measured between the two metabolic monitoring devices. A low absolute agreement was defined as a difference in RMR > 628 kJ as this would result in a significant difference in the recommended energetic consumption for an individual^(34)^. Pearson correlation was used to analyse the overall correlation at the group level in RMR and VO_2_ between the two metabolic monitoring devices. A correlation coefficient (*r*) between 0·3 and 0·5 was considered low, 0·5 and 0·7 moderate and 0·7 and 0·9 high^(35)^. Relative agreement in RMR and VO_2_ between the two metabolic monitoring devices was addressed by intraclass correlation coefficients (ICC) which were calculated using a two-way mixed model of consistency^(36)^ and interpreted as poor (below 0·50), moderate (0·50–0·75), good (0·75–0·90) or excellent (0·90 or higher)^(37)^.

Bland–Altman analysis was used to assess the absolute agreement in RMR and VO_2_ between the two metabolic monitoring devices and to visually display the individual dispersion patterns of RMR and VO_2_ assessed by the two metabolic monitoring devices^(38)^. Proportional bias was determined by a significant deviation of the slope of the regression line of the Bland–Altman plots for RMR and VO_2_ between the two metabolic monitoring devices^(39,40)^. Since proportional bias was found, differences in log-transformed RMR and VO_2_ and the 95 % limits of agreement (LOA) (mean difference ± 1·96 sd) were presented in Bland–Altman plots. For the interpretation of log-transformed values, back transformations of the mean difference and the LOA were computed to obtain the values relating to the ratios of measurements by the two metabolic monitoring devices.^(41)^

The absolute (kJ) and relative (percentages) differences between RMR measured by Fitmate GS and predicted RMR *v*. RMR measured by QUARK RMR were computed and assessed by paired *t* tests. The percentage of participants that had an accurate, underpredicted or overpredicted RMR was calculated. RMR was considered as accurate when it was within ±10 %; underpredicted if <−10 % and overpredicted if >10 % of RMR measured by QUARK RMR^(2,4)^. The root-mean-squared prediction error (RMSE) was used to indicate the predictive performance of the model in our data set. A lower RMSE indicates a better performance of the Fitmate GS or equations in predicting the data^(4,42,43)^.

Bland–Altman analysis was also used to assess the absolute agreement in RMR between predictive equations and the reference device (i.e. QUARK RMR). Proportional bias was determined by a significant deviation of the slope of the regression line of the Bland–Altman plots for RMR between QUARK RMR and predictive equations. Since proportional bias was detected for all equations, data of RMR were log-transformed. After log-transformation of data, proportional bias still existed and therefore a regression approach for non-uniform differences was conducted^(41)^. The line of best agreement and the regression-based 95 % LOA (expected difference derived from the line of best agreement ± 1·96 × residual sd from the regression) were presented in Bland–Altman plots.

The Statistical Package for the Social Sciences (SPSS) version 21.0 was used for the statistical analyses (SPSS Inc.), and Excel (Microsoft Office Excel 2016) was used to compute RMSE. *P* values of <0·05 were considered statistically significant. Visualisation of results was performed using GraphPad Prism 5.01.

## Results

Table 1 shows the participants’ characteristics. A total of sixty-eight participants were included with a median age of 22 years old (IQR 21–32). The majority of the participants were female (72·1 %).

**Table 1.** Characteristics of participants (*n* 68) (Numbers and percentages; medians and interquartile ranges (IQR); mean values and standard deviations)

	*n*		%
Female	49		72·1
Age (years)			
Median		22	
IQR		21–32	
No. of diseases			
Median		0	
IQR		0–1	
No. of medications			
Median		0	
IQR		0–1	
Physical activity level			
High	37		54·4
Moderate	28		41·2
Low	3		4·4
	Mean		sd
Weight (kg)			
Female	57·3		7·7
Male	83·0		15·8
BMI (kg/m^2^)			
Female	21·1		2·6
Male	26·9		5·4
Skeletal muscle mass (kg)			
Female	23·2		3·2
Male	34·4		5·1
Fat free mass (kg)			
Female	42·4		5·3
Male	61·1		8·1
Fat mass (%)			
Female	25·5		7·5
Male	25·0		10·3

The median CV in VO_2_ and VCO_2_ were, respectively, 8 % (IQR 6–12) and 10 % (IQR 8–14) for QUARK RMR, and the median CV in VO_2_ was 7 % (IQR 5–11) for Fitmate GS. A steady state was reached in thirty-four and fifty participants for QUARK RMR and Fitmate GS, respectively, and twenty-nine for both QUARK RMR and Fitmate GS within the same participant. Table 2 presents the agreement of RMR and VO_2_ between the QUARK RMR and Fitmate GS. The mean difference in RMR between the QUARK RMR and Fitmate GS was 649 (sd 753) kJ/d (*P* < 0·05). There were 47 % of the participants who showed <628 kJ/d difference in RMR measured by the two metabolic monitoring devices. A high positive correlation in RMR (*r* 0·86) and VO_2_ (*r* 0·86) was found between QUARK RMR and Fitmate GS (*P* < 0·05). ICC values showed good agreements between the QUARK RMR and Fitmate GS in the measurements of RMR (ICC = 0·84) and VO_2_ (ICC = 0·84) (*P* < 0·05). When the analysis was stratified by participants with (*n* 29) and without a steady state (*n* 39), there were no significant differences between the two groups in RMR, VO_2_ and their mean differences between QUARK RMR and Fitmate GS (*P* > 0·05). Pearson correlation and ICC of RMR and VO_2_ between QUARK RMR and Fitmate GS were high (all >0·8) in both groups.

**Table 2.** Agreement of RMR and VO_2_ between QUARK RMR and Fitmate GS, stratified by participants with (*n* 29) and without a steady state (*n* 39) (Mean values and standard deviations)

	QUARK RMR		Fitmate GS					
	Mean	sd	Mean	sd	Mean difference*	sd	*r*	ICC
RMR (kJ/d)								
All	5799	1469	5151	1226	649†	757	0·858†	0·844†
Steady state	5724	1238	5155	1117	569†	540	0·901†	0·896†
Non steady-state	5858	1636	5146	1318	707†	887	0·840†	0·821†
VO_2_ (ml/min)								
All	200	51	177	42	23†	26	0·857†	0·843†
Steady state	198	42	177	38	21†	18	0·904†	0·899†
Non-steady state	202	56	177	45	25†	31	0·838†	0·818†

*r*, correlation; ICC, intraclass correlation coefficient.

* RMR or VO_2_ measured by the QUARK RMR minus RMR or VO_2_ measured by the Fitmate GS.

† All statistically significant (*P* < 0·05).

**Fig. 1.**
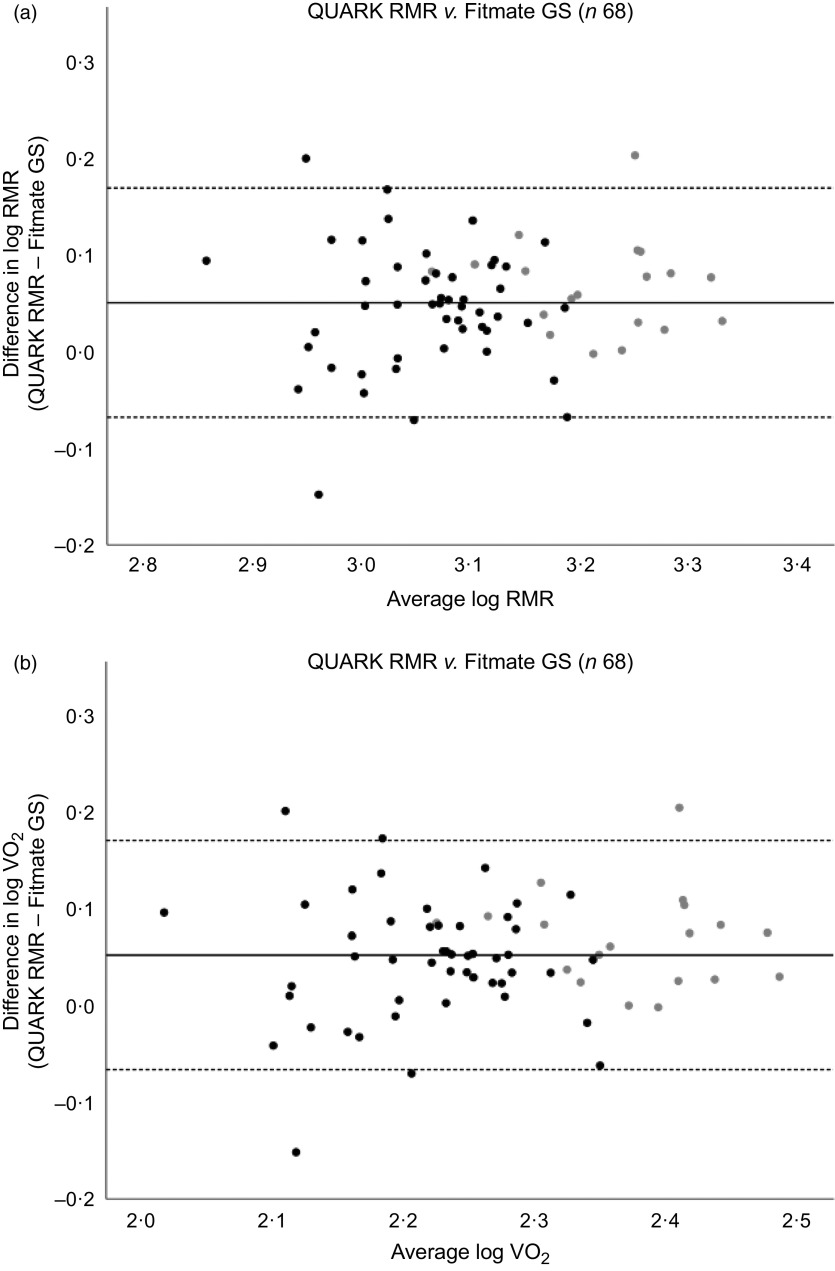
Bland–Altman plots of the difference in log-transformed (a) RMR *v*. average RMR; (b) VO_2_ *v*. average VO_2_. The solid line represents the mean difference in log-transformed (a) RMR and (b) VO_2_ measured by QUARK RMR and Fitmate GS (QUARK RMR minus Fitmate GS), while the dashed lines represent the upper and lower 95 % limits of agreement (mean difference ± 1·96 sd). 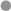, Male; 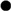, female.

Bland–Altman analysis showed significant proportional bias for RMR and VO_2_ (*P* = 0·005) indicating that the difference between the two metabolic monitoring devices was dependent on the RMR and VO_2_ measurement values (online Supplementary Fig. S1). Fig. 1 shows the Bland–Altman plots after log transformation of RMR and VO_2_. After back transformation, a mean ratio of RMR measured by QUARK RMR and Fitmate GS was 1·12 with 95 % LOA from 0·85 to 1·47 times, that is RMR measured by QUARK RMR differed from the RMR measured by Fitmate GS by between −15 % and +47 %. There were five (7·4 %) participants who had a difference in RMR and VO_2_ measured by QUARK RMR and Fitmate GS outside the 95 % LOA.

Table 3 shows the comparison of RMR measured by Fitmate GS and predicted RMR *v*. RMR measured by QUARK RMR. Paired *t* tests showed that predictive equations significantly overpredicted RMR measured by QUARK RMR, except for the Mifflin *et al.*^(27)^, Muller *et al.*^(28)^, Owen *et al.*^(30)^ and Henry 2005a^(31)^ equations. There were 52–71 % of the participants who showed <628 kJ/d difference in RMR measured by QUARK RMR and predictive equations. On the group level, predicted RMR by Mifflin *et al.*^(27)^ was the closest to the RMR measured by QUARK RMR (mean difference: −126 (sd 808) kJ/d, *P* = 0·206).

**Table 3.** Comparison of RMR measured by Fitmate GS and predicted RMR *v*. RMR measured by QUARK RMR (Mean values and standard deviations)

Measured/predicted RMR					QUARK RMR				
		RMR (kJ)			Mean difference*		% Difference†		
	Factors included in the equation	Mean		sd	Mean	sd	Mean	sd	*P*
Fitmate GS	Not applicable	5151		1226	649	757	10	13	<0·001
Weight-based predictive equations									
Harris & Benedict^(26)^	Age, sex, wt, ht	6230		1092	–431	791	–11	18	<0·001
Mifflin *et al.*^(27)^	Age, sex, wt, ht	5925		1042	–126	808	–5	18	0·206
Muller *et al.*^(28)^	Age, sex, wt	6025		1063	–226	757	–7	17	0·016
Owen *et al.*^(30)^	Sex, wt	5657		1063	146	808	0	16	0·144
Henry 2005a^(31)^	Age, sex, wt	5945		1117	–146	741	–5	16	0·112
Henry 2005b^(31)^	Age, sex, wt, ht	6004		1042	–205	761	–6	17	0·031
Schofield^(32)^	Age, sex, wt	6025		1109	–222	757	–7	16	0·018
Schofield^(32)^	Age, sex, wt, ht	6033		1117	–230	753	–7	16	0·014
WHO^(33)^	Age, sex, wt	6092		1130	–293	736	–8	16	0·002
WHO^(33)^	Age, sex, wt, ht	6117		1096	–318	761	–8	17	0·001
FFM-based predictive equations									
Mifflin *et al.*^(27)^	FFM	5657		862	146	866	–1	17	0·174
Muller *et al.*^(28)^	Age, sex, FFM, FM	5979		1025	–180	753	–6	17	0·053
Owen *et al.*^(29)^	Sex, FFM	5460		1063	339	787	4	15	0·001

wt, Weight; ht, height; FFM, fat-free mass; FM, fat mass.

* RMR measured by QUARK RMR minus RMR measured by Fitmate GS or predicted RMR.

† (Mean difference/RMR measured by QUARK RMR) × 100 %.

Table 4 shows the accuracy, overprediction and underprediction of RMR measured by Fitmate GS and each of the predictive equations compared with the RMR measured by QUARK RMR. Thirty-eight percentage of the participants had RMR measured by Fitmate GS within 10 % difference of the RMR measured by QUARK RMR. RMR measured by Fitmate GS overpredicted the RMR measured by QUARK RMR in 6 % of the participants and underpredicted the RMR measured by QUARK RMR in 56 % of the participants. Accurate prediction of equations in comparison by QUARK RMR was found in 46–68 % of the participants. Predictive equations overpredicted the measured RMR in 15–32 % of the participants and underpredicted the measured RMR in 4–40 % of the participants. FFM-based equations did not improve the accuracy as the percentage of participants that had an accurate RMR using FFM-based equations (46–60 %) was lower than most of the weight-based equations (57–68 %). Fitmate GS had higher RMSE (992 kJ) compared with predictive equations (749–958 kJ) in our population. The Henry 2005a^(31)^ equation had the lowest RMSE of all equations investigated in this study.

**Table 4.** Accuracy, overprediction and underprediction of RMR measured by Fitmate GS and each of the predictive equations compared to RMR measured by QUARK RMR (Numbers and percentages)

Measured/predicted RMR		QUARK RMR						
		Accurate*		Over-predicted†		Under-predicted‡		
	Factors included in the equation	*n*	%	*n*	%	*n*	%	RMSE (kJ/d)
Fitmate GS	Not applicable	26	38	4	6	38	56	992
Weight-based predictive equations								
Harris & Benedict^(26)^	Age, sex, wt, ht	42	62	22	32	4	6	895
Mifflin *et al.*^(27)^	Age, sex, wt, ht	46	68	16	24	6	9	812
Muller *et al.*^(28)^	Age, sex, wt	40	59	21	31	7	10	782
Owen *et al.*^(30)^	Sex, wt	39	57	14	21	15	22	812
Henry 2005a^(31)^	Age, sex, wt	45	66	16	24	7	10	749
Henry 2005b^(31)^	Age, sex, wt, ht	45	66	18	27	5	7	782
Schofield^(32)^	Age, sex, wt	46	68	17	25	5	7	782
Schofield^(32)^	Age, sex, wt, ht	45	66	17	25	6	9	782
WHO^(33)^	Age, sex, wt	45	66	18	27	5	7	787
WHO^(33)^	Age, sex, wt, ht	45	66	20	29	3	4	820
FFM-based predictive equations								
Mifflin *et al.*^(27)^	FFM	41	60	13	19	14	21	958
Muller *et al.*^(28)^	Age, sex, FFM, FM	41	60	20	29	7	10	770
Owen *et al.*^(29)^	Sex, FFM	31	46	10	15	27	40	854

RMSE, root-mean-squared prediction error; wt, weight; ht, height; FFM, fat-free mass; FM, fat mass.

* RMR fell within ±10 % of the RMR measured by QUARK RMR.

† RMR was >10 % of the RMR measured by QUARK RMR.

‡ RMR was < –10 % of the RMR measured by QUARK RMR.

**Fig. 2.**
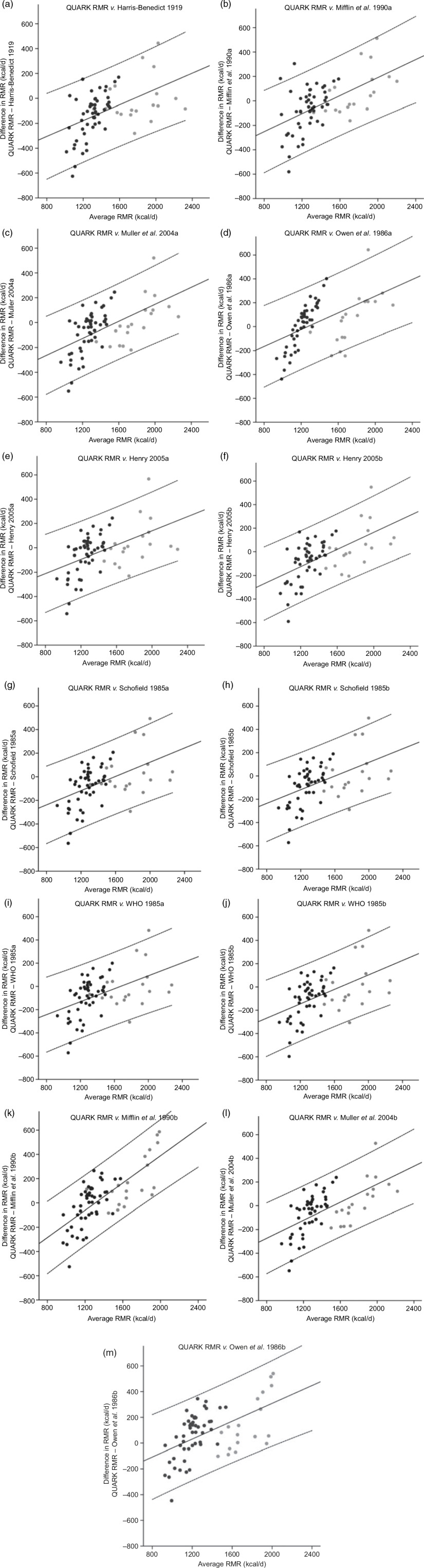
Bland–Altman plots of the difference in RMR measured by QUARK RMR and RMR predicted by equations *v*. average RMR for weight-based equations (a) Harris & Benedict^(26)^; (b) Mifflin *et al.*^(27)^; (c) Muller *et al.*^(28)^; (d) Owen *et al.*^(30)^; (e) Henry 2005a^(31)^ (f) Henry 2005b^(31)^; (g and h) Schofield^(32)^; (i and j) WHO^(33)^; and fat-free mass-based equations (k) Mifflin *et al.*^(27)^; (l) Muller *et al.*^(28)^; and (m) Owen *et al.*^(29)^. Proportional bias was observed for all equations; the solid line represents the expected difference in RMR measured by QUARK and RMR predicted by equations, while the dashed lines represent the regression-based upper and lower 95 % limits of agreement (expected difference derived from the line of best agreement ± 1·96 × residual sd from the regression). To convert kcal to kJ, multiply by 4·184. 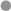, Male; 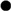, female.

Bland–Altman plots for all predictive equations compared with QUARK RMR showed proportional bias, in which equations overestimated the low RMR values and underestimated the high RMR values (Fig. 2). The 95 % LOA for the predictive equations studied were high for both weight-based equations (ranging from −1979 to +1728 kJ/d) and FFM-based equations (ranging from −1657 to +1879 kJ/d).

## Discussion

In a group of healthy adults, RMR measured by Fitmate GS was lower and RMR calculated by predictive equations were higher than those measured by QUARK RMR. A good relative agreement and a low absolute agreement were found in the measurements of RMR and VO_2_ between the QUARK RMR and Fitmate GS. Accurate RMR was found among 38 % of the participants for the Fitmate GS and among 46–68 % of the participants depending on the predictive equations. Mean RMR predicted by equations were closer to the mean RMR measured by QUARK RMR compared with those measured using Fitmate GS. Predictive equations had a low absolute agreement with QUARK RMR at the individual level due to wide LOA.

This is the first study examining the agreement in RMR measurement between the QUARK RMR and Fitmate GS within the same individuals. A previous study comparing RMR measurements in the same individuals between the QUARK CPET and Fitmate using canopy hood among thirty healthy adults aged between 18 and 45 years found a high positive correlation and excellent relative agreement in VO_2_ (*r* 0·98, ICC = 0·99) and RMR (*r* 0·96, ICC = 0·99) between the two metabolic monitoring devices^(44)^. However, Bland–Altman analysis in the same study showed no significant difference in RMR and VO_2_ measured by the two metabolic monitoring devices and LOA ranged from −1067 kJ/d to +1113 kJ/d^(44)^ compared with a significant difference of 649 kJ/d and an upper LOA of 2125 kJ/d in the present study. The wide LOA indicates a higher difference at the individual level in RMR between the two metabolic monitoring devices in the present study. The discrepancy may be explained by the different metabolic monitoring device used and/or study protocols. The RMR measurements in the aforementioned study were measured by the QUARK CPET which has different technical specifications compared with QUARK RMR and consisted of two 10-min sessions for each metabolic monitoring device and the participants remained in supine position until all measurements were completed. Data for the first 5 min were not discarded, although this is a recommendation from the best practice guideline for performing indirect calorimetry^(13)^. The longer duration of RMR measurements in this study may result in higher variation due to extraneous factors and change in the physical state^(45)^. However, current guidelines do not have a specific recommendation regarding the duration of the RMR measurement^(13)^.

The Fitmate has also been validated against the Douglas bag system among sixty healthy adults aged between 19 and 65 years^(11)^ and the ‘gold standard’ device DELTATRAC II among thirty-seven outpatients and hospitalised patients aged between 18 and 78 years, respectively^(10)^. In both studies, expired air was not collected by canopy hood and the type of Fitmate used was not specified. There were no significant differences for RMR or VO_2_ between the two metabolic monitoring devices. LOA was between −636 kJ/d and +686 kJ/d against Douglas bag^(11)^ and −1402 kJ/d and +1686 kJ/d against DELTATRAC II^(10)^. The reasons for different results may be specific to each study design, measurement protocol, gas collection device and study population. For example, RMR was measured simultaneously using the Fitmate and Douglas bag during two 10-min test sessions^(11)^. This design may minimise the variation due to extraneous factors and change in the physical state^(45)^. In the other study^(10)^, RMR was measured by Fitmate with a mask for 15 min and DELTATRAC II with canopy hood for 20 min in patients. However, it has been reported that the mask method had a 7 % higher in RMR compared with the canopy hood method among healthy individuals^(46)^.

In the present study, all RMR measurements were included in the analysis because the conclusion of the findings remains unchanged when we stratified the data by the participants with and without a steady state. This is in line with a previous study in healthy adults which suggested that participants who do not achieve steady state should not be excluded from data analysis^(47)^. There is no universal definition for steady state as it varies by measurement time, the CV and combination of gas-exchange variables (VO_2_ and VCO_2_)^(13)^. In the present study, 42·6 % of the participants reached a steady state for both QUARK RMR and Fitmate GS. Comparing the percentage of participants reaching a steady state with previous studies using either QUARK RMR or Fitmate GS is not possible because the criteria for the steady state were not defined and the percentage of participants who reached the steady state was not reported^(9–11,44)^. Studies using a canopy hood but different metabolic monitoring devices reported that 93–95 % of healthy adults reached a steady state over five consecutive minutes within the 30 min^(18,48)^. However, when stringent criteria of steady state were used (i.e. over ten consecutive minutes), only 47 % reached steady state and it may reflect increased participants’ anxiety over time^(48)^. The percentage of participants who reached steady state in our study is comparable to previous studies which used different metabolic monitoring devices, given that our steady-state criteria are more stringent, in which variations in the VO_2_ and VCO_2_ should be <10 % over an average of 25 min instead of a minimum of 4 min as accepted by the best practice guideline^(13)^.

To the best of our knowledge, there is currently no standardised protocol for measuring RMR using indirect calorimetry. For example, although the best practice guideline recommends at least 20-min rest before the RMR measurement^(13)^, recent studies among healthy young adults showed that a resting period before the RMR measurement could be omitted. In previous studies, during the 30-min RMR measurement, a steady state was achieved after 5 min and there was no difference in RMR between each of the 5-min segment after the first 5 min^(18,19)^. Therefore, in this study, a rest period prior to both the RMR measurements was not imposed in order to minimise the total duration, to create a comfortable testing environment and to mimic the practicality in clinical practice.

### Clinical implication

Metabolic monitoring devices such as QUARK RMR are known to be costly, less portable, requiring trained operators and complicated to calibrate. Because of these disadvantages, routine RMR measurement is difficult and impractical in clinical practice. Therefore, an inexpensive, easy-to-use, portable and accurate metabolic monitoring device for measuring RMR in the clinical setting is desirable^(49)^. Although QUARK RMR and Fitmate GS have been validated against the ‘gold standard’ DELTATRAC in healthy adults^(9)^ and hospitalised patients^(10)^, respectively, our findings among healthy adults showed a good agreement in RMR measurement between the QUARK RMR and Fitmate GS at the group level but a low agreement of RMR compared with QUARK RMR at the individual level. Our study also showed that RMR predicted by equations had smaller mean differences with RMR measured by QUARK RMR compared with the mean differences between RMR measured by Fitmate GS and QUARK RMR. However, it is important to note that a small mean difference between group means is not necessarily a measure of accuracy, as a high positive difference between predicted and measured RMR might counterbalance a high negative difference between predicted and measured RMR.

Our findings suggested that objective measurement of RMR should be employed if possible as predictive equations cannot account for individual variances (LOA above *a priori* set value of 628 kJ/d). Despite the findings from other studies that have reported high levels of accuracy of Fitmate GS in healthy adults^(11,44)^, our study shows that RMR measured using Fitmate GS should be interpreted with cautions since Fitmate GS underestimated RMR compared with QUARK RMR at a group level. One possible explanation for the lack of agreement between QUARK RMR and Fitmate GS could be because Fitmate GS assumes a fixed RQ of 0·85 and measures oxygen only. In the present study, a lower VO_2_ as measured by Fitmate GS compared with QUARK RMR could be the reason for underestimation of the RMR measured by Fitmate GS. Although using a metabolic monitoring device with high precision is ideal, it is also important to balance its feasibility and cost when implementing in clinical practice. Clinical judgement should be taken when determining the energy needs of an individual, and frequent monitoring of the energetic recommendation should be adopted. If both QUARK RMR and Fitmate GS are available in a setting, clinicians should use the same metabolic monitoring device for follow-up measurements for the same individual. Next to a standardised protocol in measuring RMR, the metabolic monitoring device used should preferably be the same within one cohort or when comparing between cohorts.

### Strength and limitations

This is the first study to examine the agreement between two clinically applicable metabolic monitoring devices (QUARK RMR and Fitmate GS) in one, relatively large group of healthy adults. However, this study has some limitations. Participants were relatively healthy adults, and therefore whether these findings can be extended to adults with diseases is unknown. There was a good relative agreement in RMR and VO_2_ between QUARK RMR and Fitmate GS using both canopy hoods. However, it is unclear if our findings apply to other gases collection systems such as a mouthpiece and face mask. RMR was not simultaneously measured by the two metabolic monitoring devices, which may contribute to variance in RMR between the two metabolic monitoring devices^(45)^. RMR was measured in a random order by the two metabolic monitoring devices to avoid confounding due to order effects. Furthermore, the ‘gold standard’ DELTATRAC was not available in this study. A methanol burning test for QUARK RMR performed prior to this study showed that the RQ was within the acceptable range; however, data of percentage recovery of oxygen and carbon dioxide were not available. However, it is possible that the RQ as a ratio is not affected if both oxygen and carbon dioxide are either over-recovered or under-recovered^(21)^. Nonetheless, QUARK RMR has been confirmed to be a valid metabolic monitoring device against DELTATRAC in healthy adults^(9)^. Therefore, using QUARK RMR as a reference device was justifiable.

### Conclusion

The findings of the present study demonstrate a good relative agreement and a low absolute agreement between the QUARK RMR and Fitmate GS for measurement of RMR in healthy adults. Predictive equations appear to be superior to Fitmate GS on the group level. However, there was a low absolute agreement with QUARK RMR, and therefore, the objective measurement of RMR should be encouraged. As both Fitmate GS and predictive equations had low absolute agreements with QUARK RMR at an individual level, these limitations should be considered and clinical judgement should be taken when determining the energy needs of an individual.

## Supplementary material

For supplementary material accompanying this paper visit https://doi.org/10.1017/S0007114520001014. click here to view supplementary material
