# Supplementary material for: The use of a portable metabolic monitoring device for measuring RMR in healthy adults
Source: Br J Nutr. 2020 Mar 16;124(11):1229–40. doi: 10.1017/S0007114520001014 (PMC7653515; doi:10.1017/S0007114520001014)
Supplement: Supplementary file 1 [file S0007114520001014sup001.doc]

**Supplementary Figure 1**: Bland-Altman plots of difference in (a) resting metabolic rate (RMR) versus average RMR; (b) oxygen consumption (VO_2_) versus average VO_2_.

The solid line represents the expected difference in (a) RMR and (b) VO_2_ measured by QUARK RMR and Fitmate GS, while the dashed lines represent the regression-based upper and lower 95% limits of agreement (expected difference derived from the line of best agreement ± 1.96 * residual SD from the regression). Grey dots represent males and black dots represent females.


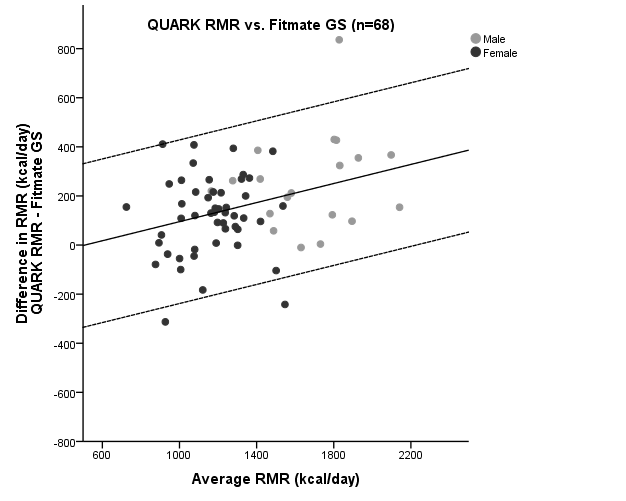
a.


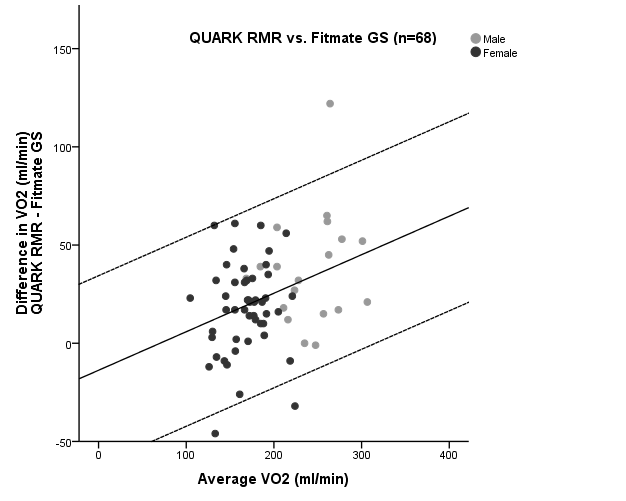
b.
